# Implicit Value Updating Explains Transitive Inference Performance: The Betasort Model

**DOI:** 10.1371/journal.pcbi.1004523

**Published:** 2015-09-25

**Authors:** Greg Jensen, Fabian Muñoz, Yelda Alkan, Vincent P. Ferrera, Herbert S. Terrace

**Affiliations:** 1 Department of Neuroscience, Columbia University, New York, New York, United States of America; 2 Department of Psychology, Columbia University, New York, New York, United States of America; 3 Department of Psychiatry, Columbia University, New York, New York, United States of America; Oxford University, UNITED KINGDOM

## Abstract

Transitive inference (the ability to infer that *B* > *D* given that *B* > *C* and *C* > *D*) is a widespread characteristic of serial learning, observed in dozens of species. Despite these robust behavioral effects, reinforcement learning models reliant on reward prediction error or associative strength routinely fail to perform these inferences. We propose an algorithm called *betasort*, inspired by cognitive processes, which performs transitive inference at low computational cost. This is accomplished by (1) representing stimulus positions along a unit span using beta distributions, (2) treating positive and negative feedback asymmetrically, and (3) updating the position of every stimulus during every trial, whether that stimulus was visible or not. Performance was compared for rhesus macaques, humans, and the betasort algorithm, as well as *Q*-learning, an established reward-prediction error (RPE) model. Of these, only *Q*-learning failed to respond above chance during critical test trials. Betasort’s success (when compared to RPE models) and its computational efficiency (when compared to full Markov decision process implementations) suggests that the study of reinforcement learning in organisms will be best served by a feature-driven approach to comparing formal models.

## Author Summary

Although machine learning systems can solve a wide variety of problems, they remain limited in their ability to make logical inferences. We developed a new computational model, called betasort, which addresses these limitations for a certain class of problems: Those in which the algorithm must infer the order of a set of items by trial and error. Unlike extant machine learning systems (but like children and many non-human animals), betasort is able to perform “transitive inferences” about the ordering of a set of images. The patterns of error made by betasort resemble those made by children and non-human animals, and the resulting learning achieved at low computational cost. Additionally, betasort is difficult to classify as either “model-free” or “model-based” according to the formal specifications of those classifications in the machine learning literature. One of the broader implications of these results is that achieving a more comprehensive understanding of how the brain learns will require analysts to entertain other candidate learning models.

## Introduction

Tests of *transitive inference* (TI) are among the oldest tools for assessing abstract thinking. First introduced by Piaget [1] to demonstrate the emergence of logic in child development, TI has since been studied in many species. The cognitive faculties that permit transitive inference are very general: To date, TI has been observed in every vertebrate species tested [2], including primates [3], rodents [4], birds [5], and even fish [6]. The widespread occurrence of this phenomenon suggests that TI procedures tap into deep and enduring learning systems.

There are obvious benefits to being able to compare scalar quantities like distance and amount, all of which are transitive by definition. Evidence also suggests that subjective evaluations of temporal duration [7] and subjective utility [8] are treated as scalar variables. For all of these characteristics, organisms will inevitably be faced with choices. Transitive comparisons avoids costly (and potentially risky) trial-and-error by allowing subjects to compare relative values following a minimal number of exposures. Provided an appropriately scalar encoding, such inferences can be achieved by direct comparison.

Some biologically relevant orderings are even more abstract, and may change rapidly. Social dominance hierarchies are an important example. Systematic analysis suggests that the vast majority of dominance relations in animals are transitive [9]. Status is often not obvious from physical appearance alone, and animals can avoid costly conflicts if they can discover and update hierarchies as third-party observers. Transitive inferences of social rank, based on observation alone, have been reported in pinyon jays [10], tilapia [8], and rhesus monkeys [11]. Furthermore, comparative studies in both corvid species [5] and lemur species [3] report a link between TI performance in a given species and the typical size of social groups in that species. Given that social groups can, in some species, consist of dozens or even hundreds of individuals, inferring social relations from partial information depends on an efficient algorithm.

**Fig 1:**
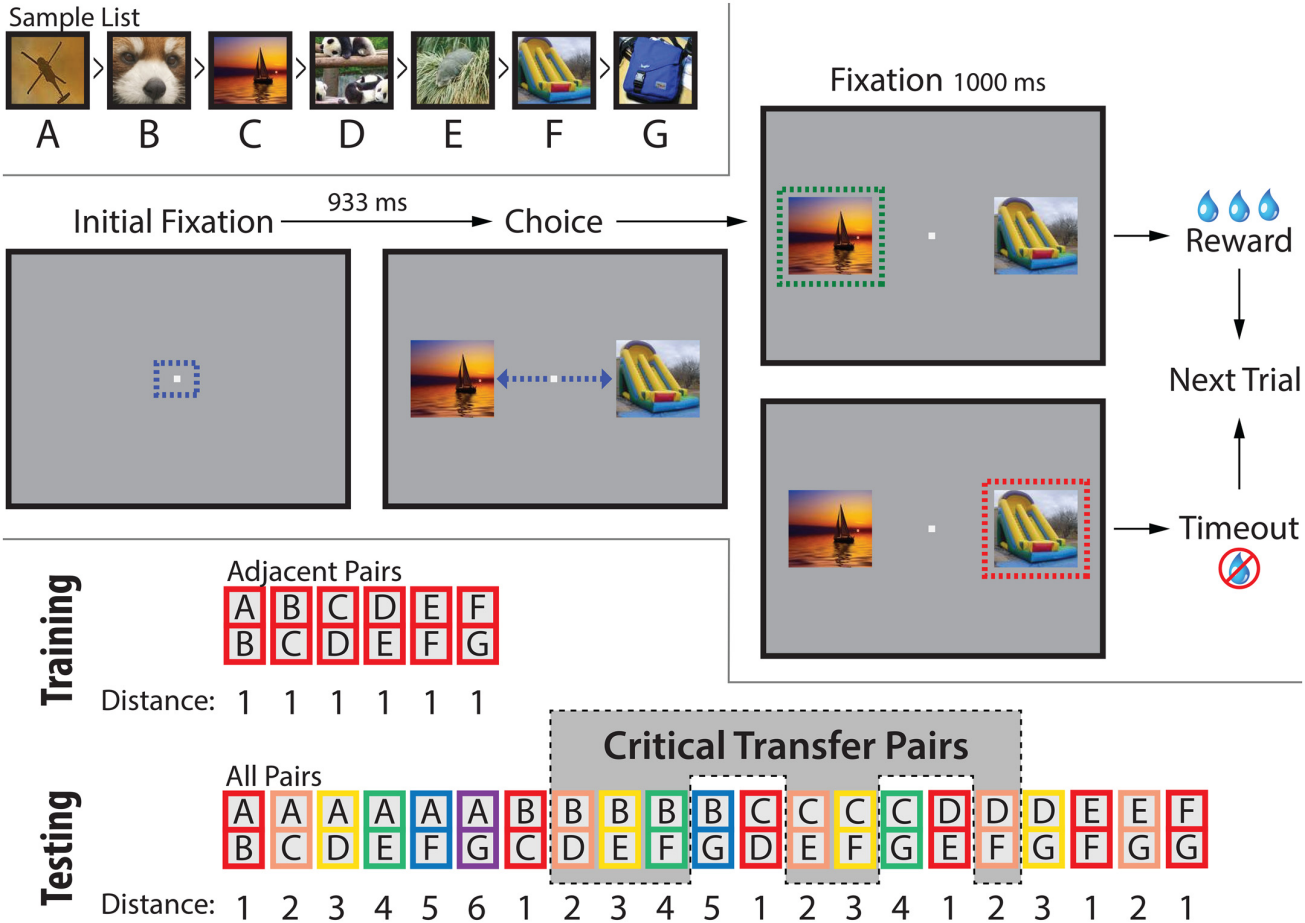
The transitive inference procedure, as implemented for rhesus macaques responding using eye tracking. (Top) Each session used a novel seven-item list, like the one depicted here. However, subjects were never presented with the entire list. (Middle) Each trial began with a fixation point. Following fixation, two stimuli appeared, and subjects received feedback upon a saccade to either stimulus. If the stimulus appearing earlier in the list was selected, a reward was delivered; if the other stimulus was selected, the animal was subjected to a timeout. Either outcome constituted the completion of a trial. In the event of an incomplete trial (e.g. subjects fixating but failing to saccade to a stimulus) was deemed incomplete and did not count toward the set of trials completed. All dashed lines and arrows represent eye movements and fixation areas, and did not appear on the screen. (Bottom) Subjects were initially trained only on the six adjacent pairs. Following adjacent pair training, subjects were then tested on all twenty-one pairs. These varied in their ordinal distance (with the pair AG being the largest). Additionally, six pairs were considered the critical transfer pairs (shaded in gray) because they were neither adjacent nor did they include the terminal items. Consequently, these are the pairs that provide the strongest test of transitive inference and symbolic distance effects.

In order to avoid confound, classical TI tasks are entirely abstract, using ordered lists assembled from otherwise arbitrary stimuli. For example, seven photographic stimuli are given the ordered labels *A* through *G*. During training, subjects are only shown randomly selected adjacent pairs (AB, BC, CD, DE, EF, and FG), and are required to select one stimulus in every trial. The only feedback provided is a reward (if the earlier list item was selected) or no reward (if the later item was selected). No other cues indicate that stimuli have an ordering, and no more than two items are ever simultaneously presented. Following training, preference is assessed for non-adjacent pairs (e.g. BD). If subjects select earlier items in novel pairs at above-chance levels, they are said to have performed a “transitive inference” because doing so exploits the transitive relationship that *B* > *C* and *C* > *D* implies *B* > *D*. Fig 1 depicts sample stimuli, trial structure, and stimulus pairings for a 7-item TI task.

In the above example, only the stimuli *A* and *G* are differentially rewarded, and can therefore be identified on the basis of reward prediction error. Accordingly, these stimuli are correctly identified more often, (the *terminal item effect*). Correct choices among stimuli *B*, *C*, *D*, *E*, and *F* are more difficult to explain, however, because their expected value during training is 0.5. The pair BD is a critical pair during testing because that pair is novel and contains no terminal items. Learning models that rely on only the expected values of stimuli fail to make the inference and respond at chance levels [12].

Despite decades of research, controversy remains over what exactly is learned during TI tasks. One such debate regards whether such learning requires cognitive processes, or can instead be explained merely by associative mechanisms. The cognitive learning school of thought holds that inferring the order of BD based only on BC and CD implies internal representation of the ordered list [13]. On the other hand, the associative learning school maintains that TI can be explained by stimulus-response-outcome associations alone [14]. Although associative models of TI struggle to accommodate the full range of empirical findings [15], their mathematical formalism at least permits specific predictions [16]. Cognitive models, by contrast, have historically been too vague to permit the simulation of behavior [12].

Here, we attempt to resolve this difficulty by comparing the ability of computational models to explain aspects of TI performance observed in humans and monkeys. These include the transfer of knowledge from adjacent to non-adjacent pairs, and symbolic distance effects. One model, drawn from the machine learning literature, can only learn from frequencies of reward delivery. Another is a new model, called *betasort*, that can infer the relative list positions of the stimuli. We argue that these models are representative of associative learning on the one hand, and cognitive learning on the other.

Humans and monkeys performed a transitive inference task, and their performance was characterized in terms of several learning models, including betasort. Betasort successfully performed the transitive inference task at low computational cost, whereas the associative model was unable to learn during adjacent pair training or to show distance effects at transfer.

## Models

### Terminology and Notation

When situating TI in the current literature, it is important to define terms. The overarching topic of *reinforcement learning* (RL) pertains to how subjects learn by trial and error, whether through associative or cognitive processes. This approach is informed by the machine learning literature (popularized by Sutton and Barto [17]), which specifies a different distinction: “model-free” RL vs. “model-based” RL [18]. Typically, these two groupings of algorithms are presented in the following fashion:

A number of accounts of human and animal behavior posit the operation of parallel and competing valuation systems in the control of choice behavior. In these accounts, a flexible but computationally expensive model-based reinforcement-learning system has been contrasted with a less flexible but more efficient model-free reinforcement-learning system.

Otto and colleagues, 2013, p. 751 [19]

With few exceptions, the “model-based” algorithms used by computational neuroscientists rely on contingency tables that relate states and actions. This represents a vast range of potential models, which either are or seek to approximate the behavior of *Markov decision processes* (MDPs). “Model-free” algorithms are in turn typically assumed to have the following characteristics:

1. Each action is represented by an expected value of reward.
2. Values are updated as a function of discrepancy between the expectation and outcome, called *reward-prediction error* (RPE).
3. Predictions are made about available actions, so only values associated with available actions are updated.

Such algorithms can solve certain problems without contingency tables, instead using RPE to approximate the expected value of a given action. These ‘value function approximations’ often rely on dynamic programming techniques pioneered by Bellman [20]. These estimated values converge at the limit with the stochastic expectations of MDP models under certain conditions. When conditions are good for rapid convergence, RPE models give rise to adaptive behavior without instantiating a contingency table [21].

Defining ‘available actions’ in a clever fashion permits RPE models to generalize. For example, by recognizing that pressing a button with one’s left or right hand may be functionally equivalent, an algorithm can learn the general predicted value of ‘button pressing’ independent of which hand is used. The most powerful such generalizations yet observed are those of “deep *Q*-network” (DQN) learning [23], which performs well under many (but by no means all) testing conditions. Other value function approximations are sometimes labeled as “model-free,” such as the Rescorla-Wagner model [22]. To avoid misunderstanding, we henceforth will refer to MDP and RPE models directly, rather than refer to broad categories of algorithms.

Although value function approximation can produce successful behavior in many contexts, it routinely fails to yield effective solutions to TI problems. Because each ‘state’ (i.e. the pair of stimuli currently visible) is independent of the previous action, and because the stimuli themselves are assigned a rank arbitrarily, there are no explicit cues that RPE algorithms can use to enhance their predictions about the expected value of the non-terminal items. Furthermore, because subjects are only told whether a response was ‘correct’ or ‘incorrect’ (as opposed, for example, to being told the distance between items following every trial), no additional information is provided about the relationship between stimuli.

Let *ch*_*t*_ denote the index associated with a subject’s choice at time *t*. Let *r*_*t*_ indicate the delivery of a reward (or lack thereof), indicated by a value of 1.0 or 0.0 respectively. Let **ℵ** denote the set of all stimuli presently employed in the experiment. **ℵ**+_*t*_ denotes only those stimuli that are presented during the current trial, while **ℵ** −_*t*_ denotes those stimuli whose presence is implied by past experience but are not currently visible. Additionally, let *nc*_*t*_ denote the set of stimuli not chosen. The models here described also make a distinction between an *updating policy* (which modifies memory as a function of feedback) and a *choice policy* (which selects the next behavior). These are best understood as subroutines.

### The Betasort Algorithm

#### Overview

Betasort is designed to be a computationally inexpensive formalization of the *spatial coding hypothesis* [13, 24–27]. By coding stimulus position spatially, betasort can perform inferences over arbitrarily large sets of items. By treating item position as a density function, rather than a point estimate, the uncertainty associated with a position can also be represented. The feedback provided during the TI task is used to shift and consolidate those stimulus densities.

**Fig 2:**
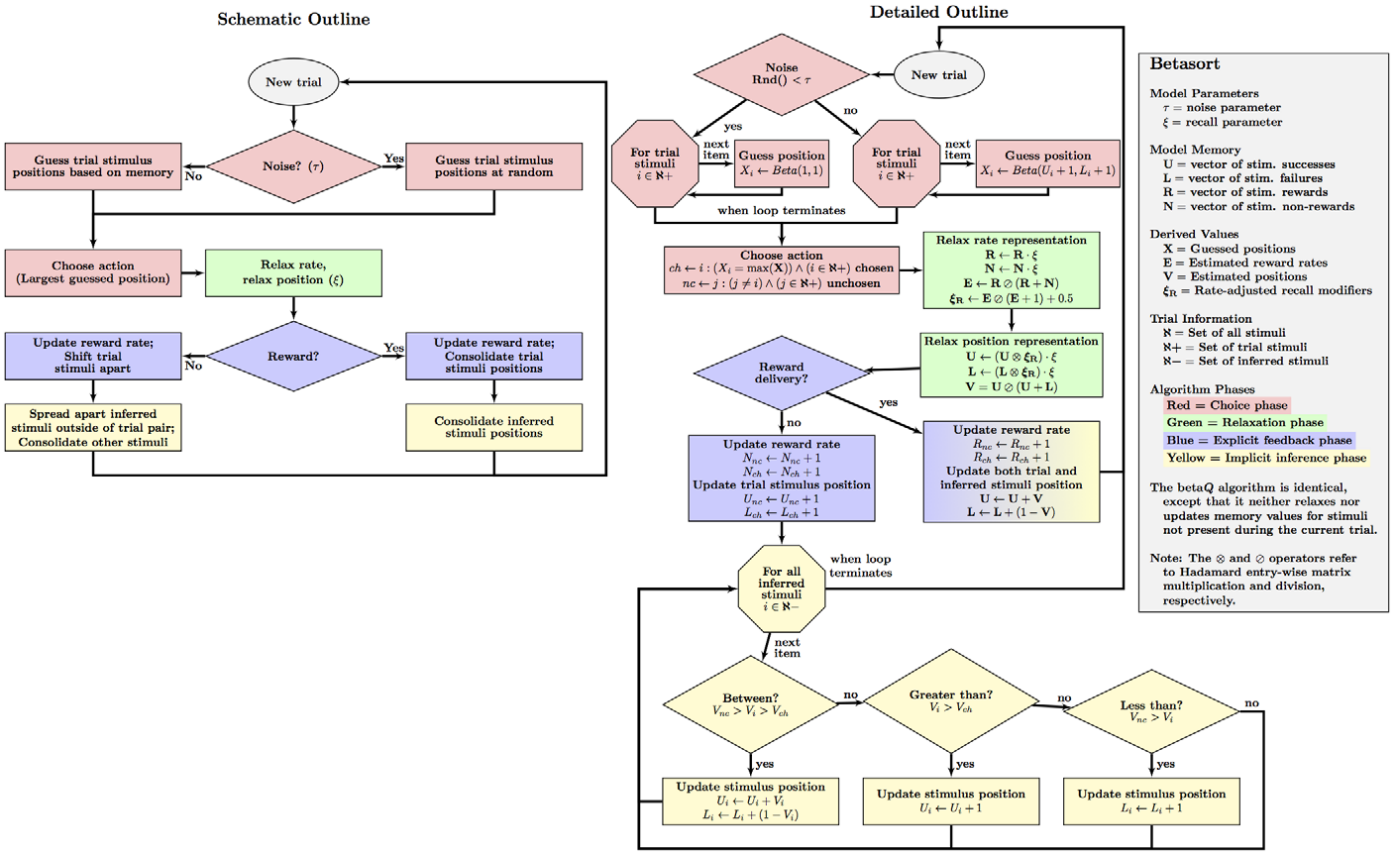
Outline of the betasort algorithm over the course of one trial. The algorithm’s logic is presented in both a schematic (left) and detailed (right) outline. Rectangles refer to operations, diamonds to logical branches, and octagons to loops that iterate over sets of items. Four phase are depicted: the choice policy (red), the relaxation of the contents of memory (green), the processing of explicit feedback (blue), and implicit inference (yellow).

Betasort directly instantiates a spatial model, and so bears little functional resemblance to an MDP approximator. It is instead based on three principles. The first is the use of beta distributions. Although commonly used as sampling distributions for probabilities, we instead use them here to represent stimulus position on a unit scale. Betasort selects behaviors using these distributions, and then updates stimulus positions and their uncertainty. The second principle is that feedback should be used to update the position of a stimulus, rather than its expected value. Consequently, when the outcome of an action is satisfactory, one should consolidate the current position, rather than shift it. The third principle is that every stimulus representation should be updated during every trial, regardless of which stimuli are presented. Collectively, these principles provide a plausible mechanism for transitive inference. A schematic representation of the algorithm is provided in Fig 2.

The position of a stimulus *i* is represented by two parameters: An “upper” parameter *U*_*i*_ and a “lower” parameter *L*_*i*_, both positive. If *U*_*i*_ > *L*_*i*_, then the stimulus position is closer to the top of the scale; if *L*_*i*_ > *U*_*i*_, then it is closer to the bottom. As *U*_*i*_ and *L*_*i*_ both get larger, the uncertainty associated with the stimulus position decreases. The density function over a sample space from 0.0 to 1.0 is given by:

$$Beta\left(x;U_{i},L_{i}\right)=\frac{\Gamma (U_{i}+L_{i})}{\Gamma (U_{i})\Gamma (L_{i})}x^{U_{i}-1}\;{\left(1-x\right)}^{L_{i}-1}$$ (1)

Here, Γ() represents the gamma function. When *U*_*i*_ = *L*_*i*_ = 1.0, the probability density is uniform; it grows increasingly normal as these parameters increase. In order to consolidate a stimulus position, rather than shift it, these parameters are increased by a proportion of their current value (i.e. $U_{i}\leftarrow U_{i}+\frac{U_{i}}{U_{i}+L_{i}}$ and $L_{i}\leftarrow L_{i}+\frac{L_{i}}{U_{i}+L_{i}}$). This distributes a single reward across both parameters, leaving the position intact while reducing its uncertainty.

Incrementing values of *U*_*i*_ and *L*_*i*_ is effectively Bayesian updating. The beta distribution at the time of a choice represents a subject’s *prior belief* about where the stimulus might be, based on the evidence collected up to that point. When the subject received feedback, this new evidence is factored in, changing the distribution to a *posterior belief*. The resulting posterior then acts as the prior for the subsequent trial. Although Bayesian updating of most continuous distributions is computationally expensive, the beta distribution is an exception because it is a *conjugate prior*. This means that, if our prior on an unknown probability is beta-distributed, then so too is the posterior. The parameters of this new posterior are identical to the prior values of *U*_*i*_ and *L*_*i*_, plus a small increment that corresponds to the feedback. Consequently, updating the beta prior entails almost no computational cost.

Because of this elegant property, the beta distribution is commonly used as a sampling distribution for an unknown probability, on the basis of a set of binary outcomes [28]. If *H* and *T* are thought of as the accumulated number of Heads and Tails resulting from flipping a coin, then *Beta*(*p*;*H*, *T*) yields a credible interval for the probability *p* of the next toss coming up Heads. Each additional Head or Tail is added to its corresponding count, tightening the beta distribution around the coin’s true probability of Heads. In betasort’s case, however, the aim is not to estimate an unknown probability, but rather an unknown position along the unit scale.

Betasort also tracks the reward (*R*_*i*_) and non-rewards (*N*_*i*_) associated with trials that include each stimulus. Importantly, if a trial is rewarded, the value of *R*_*i*_ is increased for both stimuli. This is because *R*_*i*_ and *N*_*i*_ control the algorithm’s explore/exploit tradeoff, increasing the variability of behavior when the current representation is not functioning effectively.

Betasort’s choice policy (red in Fig 2) draws random values from each position distribution, and selects the largest from among the available actions. The policy uses one free parameter: noise (0.0 < *τ* < 1.0), which is the probability that betasort ignores its memory and selects an action at random. When *τ* = 1.0, the algorithm is entirely stochastic; when *τ* = 0.0, all choices are governed by memory. Note that, early in learning, choices governed by memory will *also* look like guessing, because of the substantial uncertainty about each stimulus position. Consequently, *τ* is not strictly a variable that governs ‘guessing behavior,’ but rather one that governs how often the algorithm disregards the contents of memory.

Betasort’s updating policy begins with the relaxation phase, (green in Fig 2), which makes use of another free parameter: recall (0.0 < *ξ* < 1.0), which scales the contents of memory downward during every trial prior to processing the feedback for that trial. For example, if *U*_*i*_ = 20 and *L*_*i*_ = 10, then given *ξ* = 0.9, these values will be updated to (*ξ* × *U*_*i*_) = 18 and (*ξ* × *L*_*i*_) = 9, respectively. These representations are further relaxed as a function of *R*_*i*_ and *N*_*i*_: As the algorithm makes more mistakes, it discounts its representation more rapidly (and thus explores more); given fewer mistakes, it discounts more slowly (and thus exploits more).

**Fig 3:**
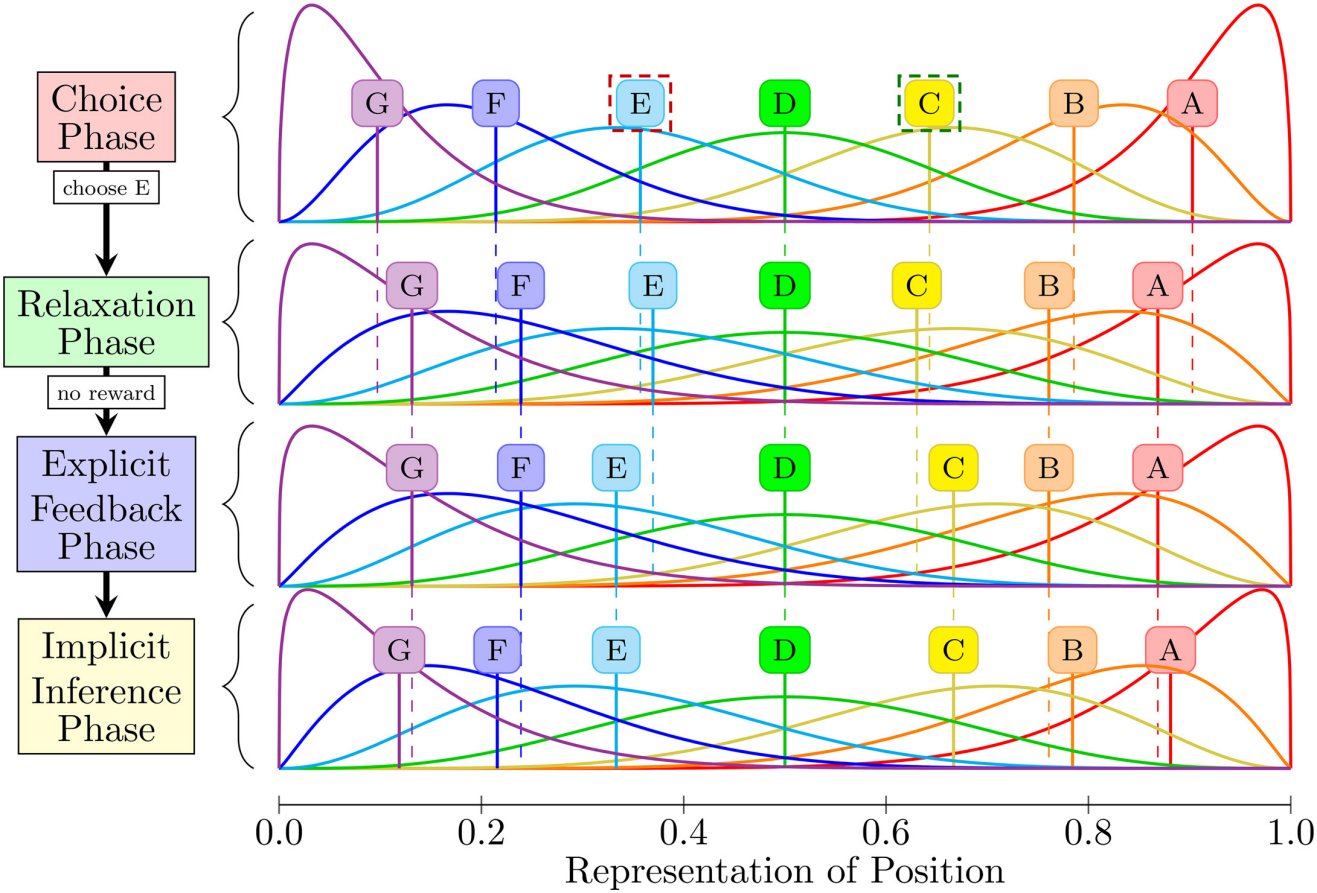
Visualization of Betasort’s adjustment of the beta distributions during a single trial in which an incorrect response is given. For this example, the trial stimuli are the pair CE. The initial conditions show the beta distributions of a well-learned list, with means marked by a vertical line. During the *choice* phase, a value is drawn randomly from the beta distributions of each trial stimulus, and the stimulus with the larger random value is chosen. In this example, the algorithm incorrectly selects stimulus *E*, an unlikely but possible event. Immediately following the choice, but before feedback is taken into account, the positions of all stimuli are *relaxed* (using *ξ* = 0.6 for this example). This has the effect of making all density functions slightly more uniform, and reduces the influence of older trials in favor of more recent ones. During *explicit feedback*, the increases *L*_*E*_ by one, while also increasing *U*_*C*_ by one. This increases the odds of future selections of stimulus *C*, while decreasing the odds of future selections of stimulus E. Next, the algorithm makes *implicit inferences* about the positions of all known stimuli that did not appear during the trial. Because stimulus *D* falls between *C* and *E*, its count of successes and failures is consolidated and its position does not change. Stimuli *A* and *B* are positioned above the trial stimuli, and so are shifted upward. Stimuli *F* and *G* are shifted downward.

Following trial feedback, betasort applies explicit feedback (blue in Fig 2) to those stimuli present in the current trial. If the choice was rewarded, both have their current positions consolidated. If the choice was not rewarded, their positions are shifted to improve performance during later trials. Next, betasort applies implicit inference (yellow in Fig 2) to the values of all stimuli not presented during the trial. If the choice was rewarded, all inferred positions are consolidated; if not, those stimuli that fall between the trial stimuli are consolidated, but those that fall outside the trial pair are shifted toward the edge of the sample space. Fig 3 presents relaxation, explicit feedback, and implicit inference during a single incorrect trial.

#### Memory structure

The betasort algorithm makes use of four 7 × 1 vectors to track feedback concerning the available stimuli: **U**, **L**, **R**, and **N**. The vector **U** indicates the degree to which stimulus i is close to the top of the unit scale. The vector **L** plays a similar role the bottom end of the scale. Jointly, **U** and **L** provide the parameters to the beta distributions that represent the estimated position of each stimulus on the unit span. Meanwhile, **R** and **N** store rewarded and unrewarded trials for each stimulus, respectively. Thus, if *R*_*i*_ = 10.5 and *N*_*i*_ = 4.5, then the algorithm estimates 70% probability of reward during trials in which i was present, based on the last 15 trials. Although all four vectors conceptually represent sums of discrete events, they support fractional values, resulting from the relaxing phase of the updating policy.

#### Choice policy

At stimulus onset for every trial, each stimulus in the set **ℵ**+_*t*_ had a number *X*_*i*_ drawn at random either from a beta distribution, parameters governed by past learning *U*_*i*_ and *L*_*i*_, or else draws these values from a uniform distribution (in which case behavior is entirely random). The odds of choosing entirely randomly is governed by the “noise” parameter *τ*, such that 0 ≤ *τ* ≤ 1:

$${\text{X}}_{i}=\{\begin{array}{cc} Beta\;\left(1,1\right) & \text{if}\;Rnd<\tau \\ Beta\;\left(U_{i}+1,L_{i}+1\right) & \text{otherwise} \end{array}$$ (2)

A value of 1 is added to *U*_*i*_ and *L*_*i*_ in order to act as a prior on the probability distribution. This also prevents the distribution from approaching a singularity as a consequence of some edge conditions during updating.

The betasort choice policy is to select the alternative whose random value is largest:

$$ch_{t}=i\;\text{such}\;\text{that}\;X_{i}=\max(X_{i\in {ℵ+}_{t}})$$ (3)

This choice policy is only marginally more expensive than softmax, in that it involves only drawing random values from a handful of closed-form probability density functions. It also has the added benefit that, because the absolute values of *U*_*i*_ and *L*_*i*_ are preserved, they govern the narrowness of the beta distributions and thus model a subject’s growing accuracy as a function of increased experience.

#### Updating policy

Betasort’s updating policy involves three stages: Relaxation, explicit updating, and implicit inference. Relaxation weakens the influence of old information in favor of more recent feedback. This is governed by the “recall” parameter *ξ*, such that 0 ≤ *ξ* ≤ 1. All four vectors (**U**, **L**, **R**, and **N**) are multiplied by *ξ*, so all qualify as “leaky accumulators” [29], steadily decreasing in absolute value. These losses are then counteracted by subsequent updating. In addition to *ξ*, the values of **U** and **L** are further relaxed by a vector of factors *ξ*_**R**_, based on the reward rate accrued during trials in which a given stimulus was present. When accuracy is high, this additional relaxation is minimal; however, when accuracy is low, more aggressive relaxation yields greater variability in behavior, helping to keep the algorithm from being trapped in local minima. Collectively, relaxation makes the following modifications, for all stimuli *i*:

$$\begin{array}{cc} R_{i} & \leftarrow R_{i}\cdot \xi \\ N_{i} & \leftarrow N_{i}\cdot \xi \\ \xi_{R_{i}} & \leftarrow \frac{\frac{R_{i}}{R_{i}+N_{i}}}{\frac{R_{i}}{R_{i}+N_{i}}+1}+0.5\\ U_{i} & \leftarrow U_{i}\cdot \xi_{R_{i}}\cdot \xi \\ L_{i} & \leftarrow L_{i}\cdot \xi_{R_{i}}\cdot \xi \end{array}$$ (4)

Subsequent updating depends on a vector of “expected values” **V** of each stimulus. These are not expected values in the econometric sense, but instead represent the best estimate of the position of each stimulus along the unit span:

$$V_{i}=\frac{U_{i}}{U_{i}+L_{i}}$$ (5)

In the event that (*U*_*i*_ = *L*_*i*_ = 0.0), *V*_*i*_ is set to 0.5. Subsequent updating depends on (1) whether the trial resulted in a reward or not, (2) whether each stimulus i was part of the set present during the trial or not, and (3) the relative values of **V**.

If the response is rewarded, then the algorithm consolidates its current estimates. This is done by increasing every *U*_*i*_ by an amount equal to *V*_*i*_, whereas every *L*_*i*_ is increased by an amount equal to (1 − *V*_*i*_):

$$\text{Choice was correct:}\;\{\begin{array}{c} U_{i}\leftarrow U_{i}+V_{i}\\ L_{i}\leftarrow L_{i}+1-V_{i} \end{array}$$ (6)

This is done regardless of whether the stimulus was present on the current trial. If, on the other hand, the response was not rewarded, then *L*_*ch*_ is increased by one, as is *U*_*nc*_. Then, for all other stimuli not present during the trial, their values are updated as a function of their *V*_*i*_ relative to the stimuli presented:

$$\text{Choice was incorrect:}\;\{\begin{array}{ll} \;U_{i\in ℵ{-}_{t}}\leftarrow & \{\begin{array}{cc} U_{i}+V_{i} & \text{if}\;V_{nc}>V_{i}>V_{ch}\\ U_{i}+1 & \text{if}\;V_{i}>V_{nc} \end{array}\\ L_{i\in ℵ{-}_{t}}\leftarrow & \{\begin{array}{cc} L_{i}+1-V_{i} & \text{if}\;V_{nc}>V_{i}>V_{ch}\\ L_{i}+1 & \text{if}\;V_{ch}>V_{i} \end{array} \end{array}$$ (7)

Thus, in the cases where the response was incorrect, the algorithm consolidates the representation of those stimuli falling between the pair, and pushes those lying outside the pair outward toward the margins.

The entire process for updating is specified by the pseudocode entitled Algorithm 1 in S1 Text.

Although this procedure involves a number of logical comparisons, its adjustments are otherwise strictly arithmetic, and can be computed rapidly without recourse to bootstrapping.

#### Parameter estimation

Although generating choices and updating memory can both be accomplished rapidly, computing the likelihood of an observed response is more computationally costly. Doing so requires computing the incomplete beta distribution:

$$I\;(x|U_{i},L_{i})=\int_{0}^{x}Beta\;(x|U_{i},L_{i})\;dx$$ (8)

In the case of a two-stimulus trial, the odds of stimulus *A* being chosen over stimulus *B*, the odds depend both on the noise parameter *τ* and integrating over two convolved distributions [30]:

$$p\;(ch_{t}=A|{ℵ+}_{t}=\{A,B\},\tau )=\frac{\tau }{2}+(1-\tau )\int_{0}^{1}Beta\;(x|U_{A},L_{A})\;I\;(x|U_{B},L_{B})\;dx$$ (9)

Given this formula, computing log-likelihoods associated with the parameters (*τ*, *ξ*) for a set of observed data can be performed in much the same manner as with the *Q*/softmax algorithm.

Unfortunately, *τ* and *ξ* are not strictly orthogonal: Performance near chance can alternatively be explained by high values for *τ* or low values for *ξ*. To avoid unstable parameter estimates, values of *τ* were estimated heuristically, based on the observation that subjects reliably showed near-ceiling performance on the pairs AF, BG, and AG. Under the assumption that the integral above equals 1.0 for those pairs, a bit of arithmetic yields the following estimate:

τ≈2-2p(correct|{A,F}∨{B,G}∨{A,G}) (10)

Having set this parameter, we then used the fminsearch() optimizer packaged with Matlab 2014b (The MathWorks, Inc.) to identify the maximum likelihood parameter estimate for *ξ*. Parameters were obtained for each session.

### The Beta*Q* Algorithm

#### Overview

To emphasize the importance of the implicit inference stage, we also present the beta*Q* algorithm, which uses beta distributions but only updates the values of stimuli present during the trial (omitting all implicit inference).

#### Memory structure

Identical to betasort: four 7 × 1 arrays, meant to represent successes **U**, failures **L**, rewards **R**, and nonrewards **N** for all stimuli.

#### Choice policy

Identical to betasort.

#### Updating policy

Incorporates only those aspects of betasort’s updating policy that relate to the presently-visible stimuli. Pseudocode describing this process is entitled Algorithm 2 in S1 Text. In this respect, beta*Q* preserves the longstanding associative assumption that only the stimuli present during the current trial can have their corresponding values updated.

#### Parameter estimation

Identical to betasort, differing only in its use of the beta*Q* updating policy.

### *Q*-Learning & Softmax

#### Overview

*Q*-learning [31] is a widely-studied RPE model that estimates each action’s expected value. *Q*-learning is only unbiased if, during training, it performs every action in every context uniformly [32]. Favoring successful actions (and avoiding harmful ones) during training can catastrophically bias its estimates. Accurate convergence is only guaranteed when *Q*-learning is paired with a counterproductive “try everything” choice policy, a recognized shortcoming of RPE models generally [33]. Despite this, *Q*-learning is often paired with the softmax function [34], a choice policy that selects actions stochastically as a function of expected values. We subsequently refer to this pairing as *Q*/softmax, which takes two parameters (*α* and *β*, described below).

#### Memory structure

Information about the stimuli was stored in a 1 × 7 vector denoted by *Q*, with each column indicating the expected value of a given stimulus on a scale from 0.0 to 1.0. By convention, the value of stimulus *i* at time *t* is denoted by *Q*_*t*_(*i*) to clearly delineate time and stimulus index. Each value of *Q*_*t*_(*i*) is initialized to a value of 0.5 when (*t* = 0). Although no *Q*-learning method is ever truly “model-free” in the cognitive sense, this constitutes the simplest model of memory that accommodates RPE-based updating.

In theoretical discussions of reinforcement learning, the value stored in memory is routinely denoted by *Q*_*t*_(*s*_*t*_, *a*_*t*_), where *s*_*t*_ refers to a current state, whereas *a*_*t*_ refers to a particular action in that state. This formalism is particularly ill-suited to the transitive inference procedure, however, because the states about which we are curious during testing (i.e. non-adjacent pairs) have never before been seen. The manner in which the model extrapolates to these hitherto-unknown states must be formally specified. Rather than smuggle inference in at the extrapolation stage, the present model is limited to ascribing an expected value to each stimulus and extrapolating on the basis of the relative values in any stimulus pairing.

#### Choice policy

Stimuli are selected using the softmax function, which has one free parameter *β*, such that *β* ≥ 0:

$$p\;(ch_{t}=i|{ℵ+}_{t},Q_{t})=\frac{\exp(\beta \cdot Q_{t}(i))}{\sum_{j\in {ℵ+}_{t}}\exp(\beta \cdot Q_{t}(j))}$$ (11)

#### Updating policy

This algorithm uses the most basic form of temporal difference reward-prediction error, which has a single free parameter *α*, such that 0 ≤ *α* ≤ 1:

$$\begin{array}{l} Q_{t+1}\left(i\right)\;=Q_{t}\left(i\right)+\alpha \delta_{t}\left(i\right)\text{, for all}\;i,\;\text{given that}\\ \delta_{t}\left(i\right)=\{\begin{array}{cc} 1-Q_{t}\left(i\right) & i\in ℵ{+}_{t}\wedge \left(\left(r_{t}=1\wedge ch_{t}=i\right)\vee \left(r_{t}=0\wedge ch_{t}\neq i\right)\right)\\ -Q_{t}\left(i\right) & i\in ℵ{+}_{t}\wedge \left(\left(r_{t}=0\wedge ch_{t}=i\right)\vee \left(r_{t}=1\wedge ch_{t}\neq i\right)\right)\\ 0 & \text{otherwise} \end{array} \end{array}$$ (12)

Thus, the value of *Q*_*t*_(*i*) for every stimulus *i* is updated at time *t*, but only those present during the current trial (i.e. *i* ∈ **ℵ**+_*t*_) are updated as a consequence of the feedback *r*_*t*_. If the choice was rewarded, the value of the chosen stimulus is increased by some factor of the discrepancy between the reward and the value, while the unchosen stimulus has its value correspondingly decreased. If, on the other hand, the choice was not rewarded, the opposite occurred: The selected stimulus was decreased and the unchosen alternative was increased. The updating process is also specified by the pseudocode entitled Algorithm 3 in S1 Text.

Note that many RPE implementations do not use this symmetrical structure, and instead only update the stimulus that was selected. We implemented both the version above and a chosen-stimulus-only updating procedure, and these yielded nearly indistinguishable results.

#### Parameter estimation

Since softmax gives the probability of selecting an outcome directly (once *β* is specified), and since the value of *Q*_*t*_(*i*) is straightforwardly defined for every trial (once *α* is specified), it is therefore easy to calculate the log-likelihood associated with a set of parameters (*α*, *β*), given an observed history of choices and responses (as described by Daw [35]). Because *Q*/softmax is an iterative algorithm, no closed-form solution exists for finding the parameters that maximum the likelihood. Consequently, these were identified using the fminsearch() optimizer packaged with Matlab 2014b (The MathWorks, Inc.). In general, optimal parameters of *β* were large (i.e., greater than 4), in order to guarantee the stimulus with the greater value was selected almost exclusively. This, in turn guaranteed values of *α* that were very small, to prevent preferences becoming too extreme too quickly.

## Results

Here, we present an analysis of TI performance by rhesus macaques and college undergraduates, the raw data of which are provided in S1 Dataset. Additionally, these empirical results are modeled using three algorithms: betasort, beta*Q*, and *Q*/softmax.

### Behavioral Results from Rhesus Macaques

Three rhesus macaques completed sessions of TI training, learning novel 7-item lists during every session. Choices were made using eye movements. The six adjacent pairs (AB, BC, …, FG) were presented in randomized blocks of twelve pairs each to counterbalance for stimulus position. After 20 blocks of training, subjects were presented with all 21 pairs of stimuli in a similarly counterbalanced fashion.

**Fig 4:**
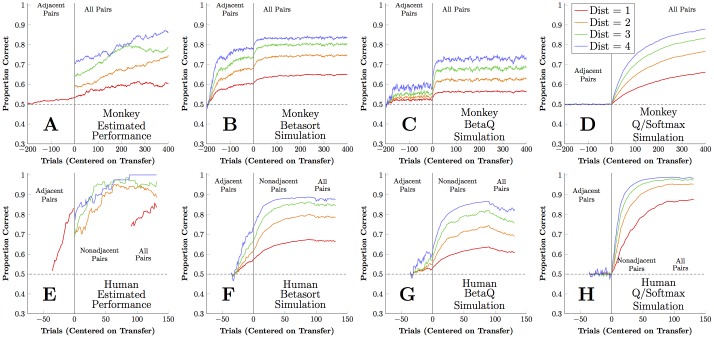
Monkey and human performance on non-terminal stimulus pairs. Trial zero is the point of transfer from adjacent-pair to all-pair trials. (A) Smoothed response accuracy for three rhesus macaques, divided into pairs with ordinal distance one (BC, CD, DE, and EF; red), two (BD, DE, and CF; orange), three (BE and DF; green), and four (BF; blue). Subjects show an immediate distance effect from the first transfer trial. (B) Simulated performance using betasort, using each monkey’s maximum-likelihood model parameters for each session. Hypothetical performance is plotted for all distances at all times, to show how the algorithm would respond had it been presented with trials of each type. Like the monkeys, the algorithm displays an immediate distance effect. (C) Simulated performance using beta*Q*, with maximum-likelihood parameters. Although a small distance effect is observed, performance remains close to chance throughout training. (D) Simulated performance using *Q*/softmax. Performance remains strictly at chance throughout adjacent-pair training, and only begins to display a distance effect after the onset of the all-pairs trials. (E) Performance of human participants given 36 trials of adjacent-pair training, followed by 90 trials of non-adjacent pairs only, and finally 42 trials of all pairs. Unlike the monkeys, participants rapidly acquire the adjacent pairs, and show only a mild distance effect at transfer. (F-H) Simulations based on human performance using the three algorithms, analogous to panels B through D. As in the monkey case, *Q*/softmax displays no distance effect at all until non-adjacent pairs are presented.

Fig 4A shows response accuracy (averaged across monkeys) for the non-terminal adjacent pairs (BC, CD, DE, and EF, in red), as well as the critical pairs with ordinal distance 2 (BD, CE, and DF, in orange), 3 (BE and CF, in green), and 4 (BF, in blue). Adjacent-pair performance is close to chance during training, but performance is above chance on non-adjacent pairs at transfer, showing a symbolic distance effect (with highest accuracy for distance 4 pairs, followed by distance 3, etc.). This constitutes a symbolic distance effect [25, 36], which is consistent with subjects’ use of serial representations. Over the next 400 trials of training, performance continued to improve. Each algorithm had two free parameters (noise *τ* and recall *ξ* for betasort and beta*Q*; *α* and *β* for *Q*/softmax) that were fit to the monkey data using a maximum likelihood method, as described in the methods. The sequence of stimulus pairs shown during each session was then presented to each algorithm using that session’s best-fitting parameters. Simulated performance was then averaged to compare the algorithms to the monkeys.

Fig 4B shows the average simulated performance of the betasort algorithm, based on the sessions and best-fitting parameters derived from the monkey data. Because simulation permits undisruptive probe trials, putative accuracy for non-adjacent pairs is also plotted during adjacent-pair training. Although monkeys and betasort differ in their particulars, several important hallmarks of the TI behavior are displayed. A distance effect is observed with the non-adjacent critical pairs, which persists over the course of the subsequent training. Contrastingly, Fig 4C shows the average simulated performance of the beta*Q* algorithm, which displays less resemblance to the monkey data. Although a weak symbolic distance effect is observed, it does not exceed an accuracy of 60% at transfer. Fig 4D, which displays the average simulated performance for the *Q*/softmax algorithm, resembles the monkey data the least: Its performance on the non-terminal adjacent pairs is precisely 50% throughout training, and no transitive inference is displayed at transfer. Instead, the algorithm begins the all-pairs phase of the experiment at chance on all critical pairs, and only gradually determines their ordering once it has received all-pair training.

**Fig 5:**
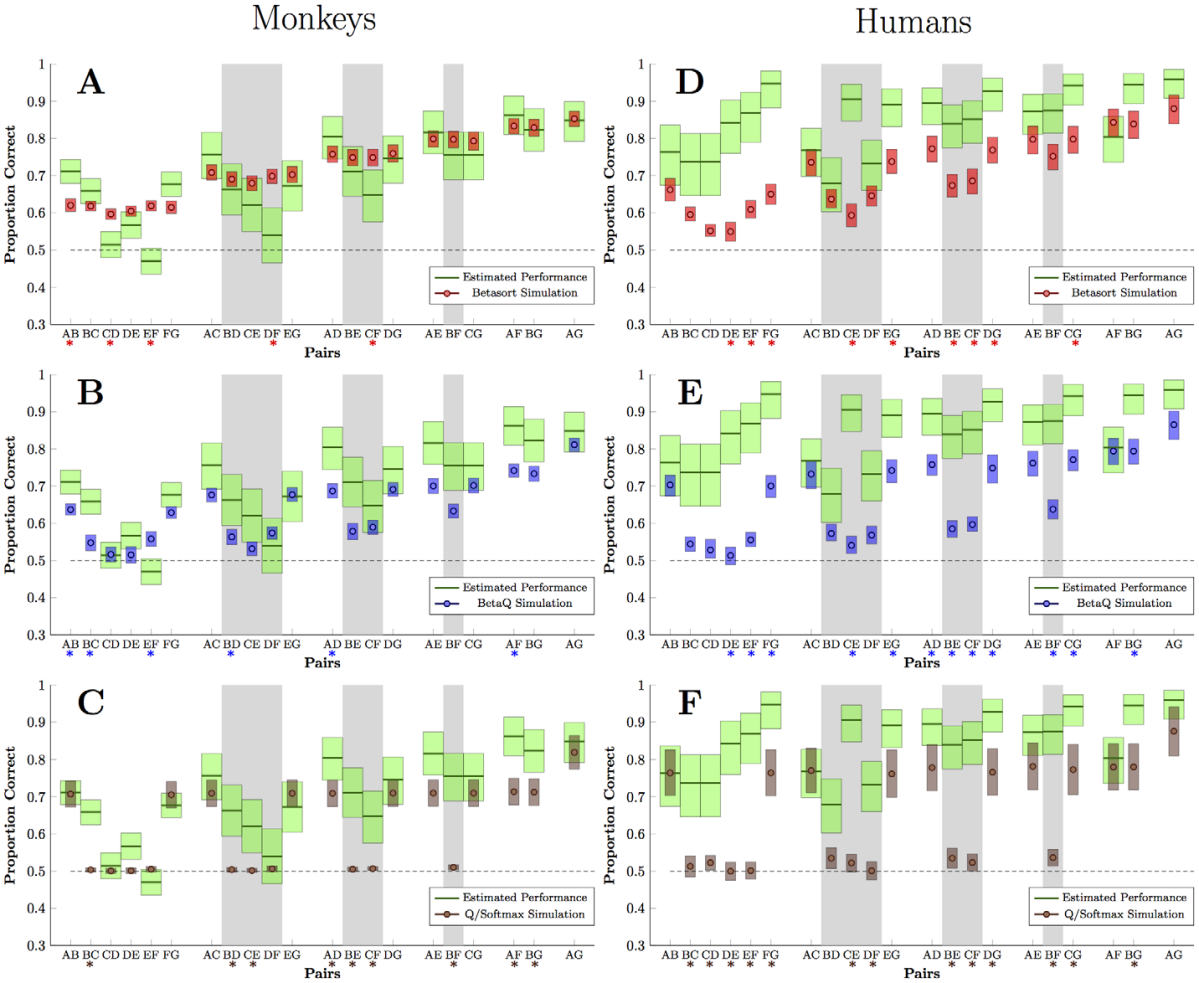
Estimated response accuracy on the first transfer trial for each of the 21 possible pairs. Estimates compare performance by subjects (blue lines) to those generated by simulations using each algorithm. Those pairs with a gray backdrop are the critical transfer pairs that are not expected to be subject to the terminal item effect. Shaded regions around each point/line represent the 95% confidence interval for the mean, determined using bootstrapping. (A) Monkey performance (green) compared to the performance of the betasort algorithm (red), given each session’s maximum likelihood parameter estimates. An overall distance effect is reliably observed from the simulation. (B) Monkey performance (green) compared to the beta*Q* algorithm (blue), given maximum likelihood parameters. Although a distance effect is evident among critical pairs, beta*Q* fails to perform appropriate levels of accuracy. (C) Monkey performance (green) compared to the *Q*/softmax algorithm (brown), given maximum likelihood parameters. Apart from a robust terminal item effect, the algorithm’s responding is strictly at chance, including all critical transfer pairs. (D-F) Human performance compared to the three algorithms, analogous to panels A through C. Although none of the algorithms precisely resemble the participants, the betasort algorithm comes closest, yielding a distance effect on critical transfer pairs.

Fig 5A–5C presents the contrast between model predictions and animal behavior during the first block of trials following transfer. The betasort algorithm, in red, (Fig 5A) largely aligns with observed response accuracy, in green, for all 21 possible pairs. This includes a distance effect for the critical pairs, which are indicated by a gray backdrop. Confidence intervals were calculated using bootstrapping, and corrected for multiple comparisons using the Holm-Bonferroni step-down procedure [37]. Those pairs whose means differ significantly are denoted in Fig 5 with an asterisk below the pair’s axis label; 5 such pairs differed significantly in Fig 5A. Contrastingly, the beta*Q* algorithm (Fig 5B, blue) did less well in approximating performance. While the betasort algorithm tended to overperform on the critical transfer pairs, beta*Q* tended to underperform. Of the 21 pairs, beta*Q* differs significantly from observed performance for 6. The *Q*/softmax algorithm (Fig 5C, brown) transfered poorly: Its goodness of fit was driven by terminal item effects, and all non-terminal pairs displayed chance performance. Of the 21 pairs, 9 differed significantly from observed.

Two omnibus model comparisons of algorithm performance at transfer were calculated for each model, relative to subject accuracy: the Schwarz-Bayes Information Criterion [38] (“SBIC”) and the log-likelihood ratio [39] (“G”). Betasort received the lowest (i.e. most favorable) score in both cases (SBIC = 9248.7, G = 186.5) compared to beta*Q* (SBIC = 9264.8, G = 200.7) and *Q*/softmax (SBIC = 9338.6, G = 276.4). This constitutes strong evidence favoring betasort over the two competing models. Since SBIC provides an approximation of twice the log marginal model likelihood, it may also be used to compute an approximate Bayes factor [40]. According to this metric, the evidence strongly favors betasort over both beta*Q* (log_*e*_(*BF*) = 8.1) and *Q*/softmax (log_*e*_(*BF*) = 45.0).

### Behavioral Results from Humans

19 college undergraduates completed sessions of TI training, using a touchscreen. Training consisted of 36 trials consisting of adjacent pairs, which was then followed by 90 trials consisting of only the non-adjacent pairs. The session then concluded with 42 trials using all pairs. Average response accuracy for the non-terminal pairs is shown in Fig 4E, for four symbolic distances.

As with the monkeys, best-fitting algorithm parameters were identified based on maximum likelihood, and these were used to simulate the behavior of each participant. Fig 4F–4H depict average performance of the simulations at transfer. Although betasort shows the closest approximation of the symbolic distance effect at transfer, several discrepancies are evident. In particular, performance on the non-terminal adjacent pairs is rapidly learned by humans, but is not learned by the algorithms. This suggests that, in addition to an inferential procedure, participants also used rote memorization.

Fig 5D–5F show performance at transfer for each of the 21 pairs by humans (green) vs. each of the algorithms. Betasort (red) provided the best fit: 9 pairs differed significantly at transfer, based on bootstrapped comparisons of means corrected for multiple comparisons, compared to 12 and 13 pairs for beta*Q* (blue) and *Q*/softmax (brown) respectively. As in the monkey case, the *Q*/softmax algorithm shows no symbolic distance effect at transfer. The same omnibus model comparisons performed for the monkeys were also computed for the humans, and these also favor betasort (SBIC = 1001.1, G = 152.1) over beta*Q* (SBIC = 1062.3, G = 213.4) or *Q*/softmax (SBIC = 1100.1, G = 251.3). In direct models comparisons using approximate Bayes factors, betasort is favored over both beta*Q* (log_*e*_(*BF*) = 25.6) and *Q*/softmax (log_*e*_(*BF*) = 44.5).

### Simulations

Betasort provided a better fit to the human data than beta*Q* or *Q*/softmax, but all three displayed poor transfer on critical pairs. This poor fit might reflect the model’s inability to do well in general, or 36 trials may not be sufficient training. To assess this, each algorithm was presented with extended adjacent-pair training in order to determine how rapidly transitive inference effects were expected to emerge.

**Fig 6:**
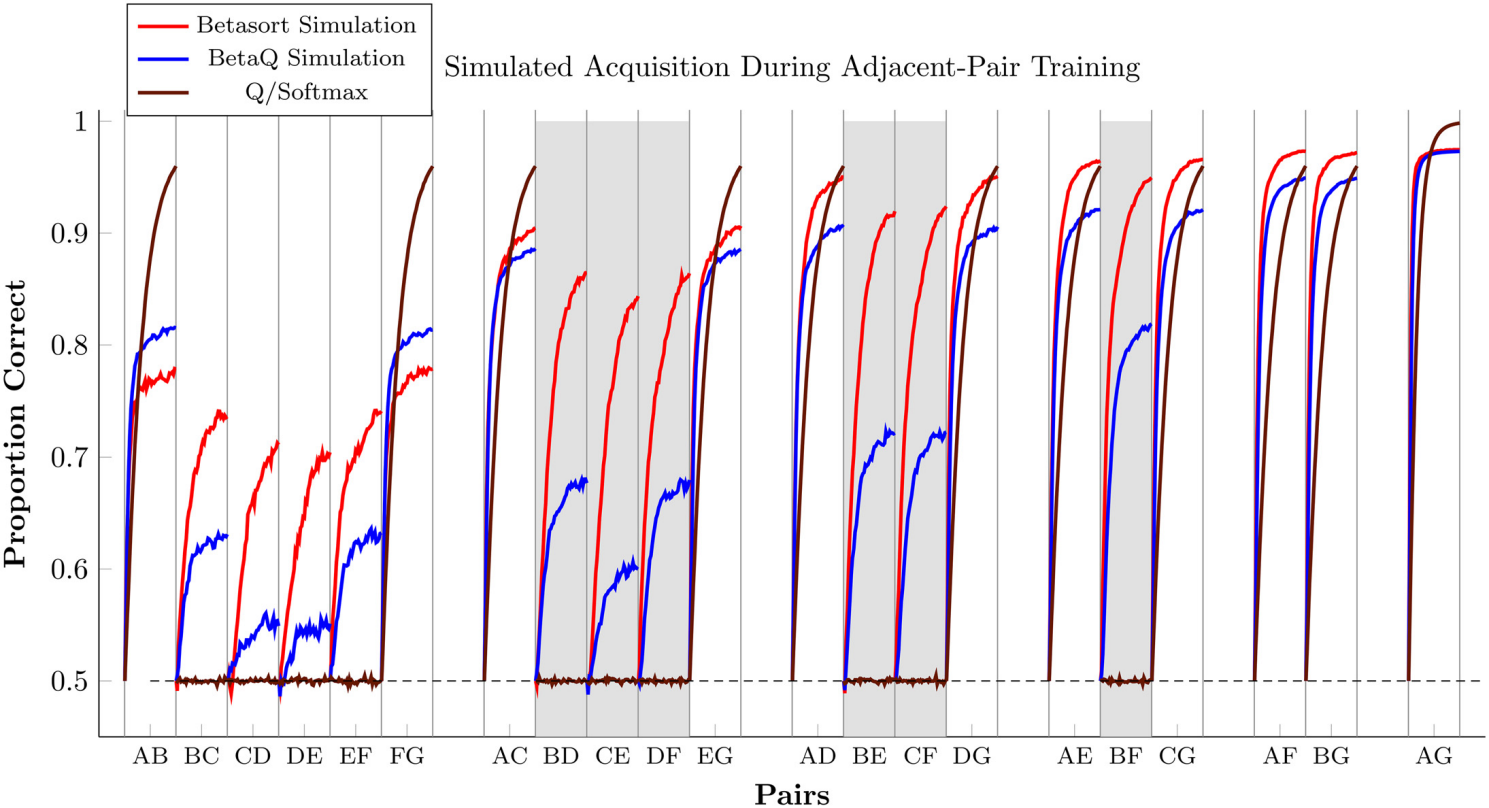
Simulated response accuracy for all stimulus pairs over the course of 200 trials of adjacent-pair training. Performance was modeled using betasort (red), beta*Q* (blue), and *Q*/softmax (brown). Critical transfer pairs are indicated with a gray shaded background. Both betasort and beta*Q* used the same parameters (*τ* = 0.05, *ξ* = 0.95), while *Q*/softmax used the parameters (*α* = 0.03, *β* = 10). Betasort shows more pronounced transfer in the critical pairs, whereas beta*Q* shows a more pronounced terminal item effect. *Q*/softmax rapidly acquires the terminal items, but remains strictly at chance for all non-terminal pairs.

Fig 6 displays response accuracy, for each pair, over 200 trials of adjacent-pair training. Using parameters similar to those obtained from the highest-performing human participants (*τ* = 0.05, *ξ* = 0.95), the betasort algorithm (red) rapidly improved accuracy for all non-adjacent items, exceeding 80% accuracy for critical transfer pairs after 200 trials. The beta*Q* algorithm (blue), working with the same parameters, fared worse, but nevertheless showed a symbolic distance effect. *Q*/softmax (brown; *α* = 0.03, *β* = 10) remained at precisely chance levels for all non-terminal pairs.

**Fig 7:**
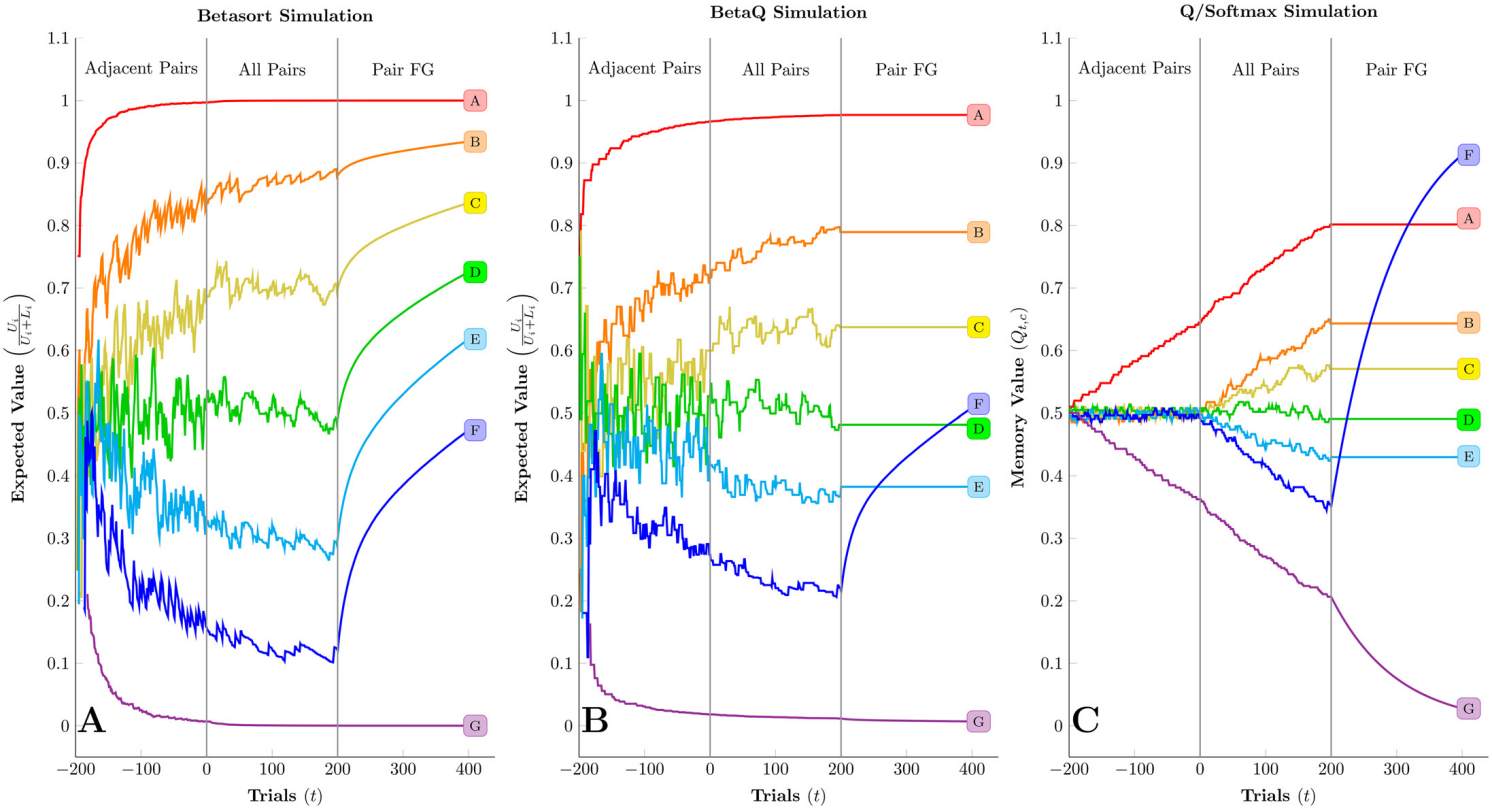
Visualization of the contents of memory for the three algorithms under simulated conditions. Three phases were included for each algorithm: 200 trials of adjacent pairs only, followed by 200 trials of all pairs, and then followed by 200 massed trials of only the pair FG. (A) Expected value for each stimulus under the betasort algorithm, given parameters of *τ* = 0.05 and *ξ* = 0.95. Not only is learning during adjacent pair training faster, but massed trials of FG do not disrupt the algorithm’s representation of the order, because occasional erroneous selection of stimulus *G* increases the value of all stimuli, not just stimulus *F*. (B) Expected value for each stimulus under the beta*Q* algorithm, given parameters of *τ* = 0.05 and *ξ* = 0.95. Although the algorithm derives an ordered inference by the time the procedure switches to all pairs, that order is not preserved during the massed trials of FG, as a result of the lack of inferential updating. (C) Expected value *Q* for each stimulus under the *Q*/softmax algorithm, given parameters of *α* = 0.03 and *β* = 10. Values for non-terminal items remain fixed at 50% throughout adjacent pair training, and only begin to diverge when all pairs are presented in a uniformly intermixed fashion. Subsequent massed training on the pair FG disrupts the ordered representation because rewards drive the value of stimulus *F* (and the value of stimulus *G* down) while the other stimuli remain static.

To showcase the trial-by-trial behavior of each algorithm, another simulation was performed, consisting of three phases: (1) 200 trials of adjacent pairs only, (2) 200 trials of all pairs, and then (3) 200 pairs of only the pair FG. This third phase was included to test the prediction that inferential updating should make betasort’s representation of stimulus positions robust against massed trials (unlike RPE models, which are expected to fail [12]). Rather than response accuracy, Fig 7 depicts the contents of memory for each of the algorithms ($\frac{U}{U+L}$ in the case of betasort and beta*Q*, and *Q* in the case of *Q*/softmax; full density functions are omitted for clarity).

Fig 7A shows expected values for the betasort algorithm. By the end of adjacent pair training, betasort has inferred that items should be spaced evenly over the unit span. Subsequent massed trials do not disrupt the stimulus ordering. Although the value of stimulus *F* rises, implicit inference ensures that stimuli *A* through *E* are modified accordingly.

Fig 7B shows the expected values for the beta*Q* algorithm. Although a mild symbolic distance effect is observed at transfer, non-terminal items remain clustered near the center. During the third phase, massed pairings of FG causes the expected value of stimulus *F* to move out of order. Because the stimuli *A* through *E* are not presented during this period, their values remain static.

Fig 7C depicts the stored memory for the *Q*/softmax algorithm. During training, the values of all non-terminal *Q*_*i*_ remain at 0.5, which is why the algorithm fails at transitive inference. The ordering begins to emerge when all pairs are presented in the second phase, but is disrupted by massed FG trials.

### Computational Complexity

Although betasort appears diagrammatically elaborate in Fig 2, the calculations it performs are generally inexpensive, and its performance scales linearly as a function of trial complexity. This linear computational cost is the basis for our claim that betasort is computationally inexpensive. Furthermore, betasort’s memory load is also trivial.

Let *N* represent the number of stimuli in a list used in a serial task, and let *M* represent the number of stimuli presented per trial (such that *N* ≥ *M*). The largest memory structure needed for either betasort of beta*Q* to perform the operations is a *N* × 4 matrix, with an additional two variables needed for model parameters, an *N* × 2 matrix needed for model-related calculation of **X** and **V**, and a half-dozen or so variables local to the random sampling aspect of the Choice phase. A rigorous accounting also includes *N* flag variables to distinguish between present vs. absent stimuli. Thus, it is a good rule of thumb to say that all betasort variables may be fit into an *N* × 8 matrix, regardless of the size of *M*.

The procedure outlined in Fig 2 specifies only four artihmetic operations (addition, subtraction, multiplication and division) and inequality comparators. The procedure also draws random samples from the beta distribution, a process that can be performed efficiently using a procedure outlined by Press and colleagues [41]. To assess the computational cost, let *γ* represent the fixed cost associated with a particular operation, which (for the sake of concision) we will group into *γ*_ar_, *γ*_cmp_, and *γ*_beta_, respectively. Of these *γ*_beta_ is unambiguously the most costly, requiring about 20 times as long to run as would sampling from a uniform distribution. We assume, in this breakdown, that set membership evaluation can be encoded efficiently using inequality comparators.

The cost, then, of the worst case scenario for betasort would have an approximate cost of:

$$\left(2\gamma_{\text{ar}}+3\gamma_{\text{cmp}}+\gamma_{\text{beta}}+C_{M}\right)M+\left(16\gamma_{\text{ar}}+3\gamma_{\text{cmp}}+C_{N}\right)N+C_{all}$$

Here, each *C* is a constant that absorbs fixed costs such as value assignments resulting from comparisons. Of these, the largest single cost is due to the uniform random sample compared to *τ*. Since all arithmetic operations use item-wise multiplication, and since only a handful of comparator operations are needed per item, the runtime for betasort is *O*(*M*+*N*), which reduces to *O*(*N*) in the case that *N* = *M*. In practice, since most studies use *M* = 2, the expensive beta sampler is nearly a fixed cost.

To demonstrate that runtime is approximately linear (and objectively fast), simulations were performed using Matlab 2014b (MathWorks Inc.), running on a last-2013 Macbook Pro with a 2.6 GHz Intel Core i7 processor. Each simulated ‘session’ consisted of 1,000 trials. Sessions were run for various values of *N* (up to 625) and *M* (up to 47). The results of this simulation are presented in Table 1. Based on these estimates, typical runtime can be approximated by *T* = 0.0217*M* + 0.0006*N*+0.5133 (in seconds), revealing that *M* is the dominant variable, presumably due to *γ*_beta_.

**Table 1:** Median runtime of 1000 trials of betasort, as a result of 1000 simulations.

	*M* = 2	*M* = 3	*M* = 5	*M* = 7	*M* = 11	*M* = 47
*N* = 4	0.53 s	0.55 s	N/A	N/A	N/A	N/A
*N* = 9	0.57 s	0.60 s	0.64 s	0.67 s	N/A	N/A
*N* = 16	0.59 s	0.64 s	0.68 s	0.70 s	0.76 s	N/A
*N* = 25	0.61 s	0.67 s	0.72 s	0.74 s	0.80 s	N/A
*N* = 36	0.63 s	0.70 s	0.75 s	0.79 s	0.85 s	N/A
*N* = 49	0.65 s	0.71 s	0.79 s	0.83 s	0.90 s	1.81 s
*N* = 625	0.94 s	1.07 s	1.15 s	1.20 s	1.29 s	1.93 s

In principle, the computational costs of beta*Q* are also of order *O*(*M* + *N*), and are close to those of betasort. Beta*Q*’s only difference is a set of order *N* additional arithmetic and inequality comparisons performed during the implicit inference phase; they are otherwise identical, including the cost of beta sampling. In practice, differences may be observed as a function of the manner in which the code has been optimized.

## Discussion

We present two new models, betasort and beta*Q*, alongside data from humans and monkeys performing a transitive inference task. Whereas RPE models typically update the values of states leading to the current outcome, betasort updates all state values that can be inferred on the current trial. Although beta*Q* displays mild inference at transfer, betasort yields a much more pronounced distance effect that better matches the empirical behavior of subjects, particularly the monkeys (Fig 5). This is achieved through active modification of memory for implicit stimuli. Although these algorithms incur low computational cost, they are unambiguously cognitive models: They place each stimulus along a putative number line and track the uncertainty of those positions (Figs 2 and 3).

Both betasort and beta*Q* demonstrate transitive inference on critical test pairs (Figs 4 and 5), a consequence of tracking putative stimulus positions as a serial representation. Positive feedback thus consolidates current position values, even if those values are low. Even without implicit inference, beta*Q* is able to gradually ratchet its way towards reasonable values. However, implicit inference allows betasort to substantially outperform beta*Q*. Another virtue of betasort is that it protects against bias introduced by massed presentation of specific pairs (Fig 7A).

The strongest evidence favoring the serial representation hypothesis is the consistency of symbolic distance effects among critical test pairs [25], which betasort also demonstrates (Figs 4 and 5). Pairs of items with greater symbolic distance (i.e. the number of steps needed to traverse the list from one stimulus to the other) reliably yield more accurate discriminations at transfer. Thus, contrary to predictions based on expected value, subjects should not only favor stimulus B when presented with BE, but should also do so more than during BD trials. Crucially, the strength of a distance effect *at the precise moment of transfer* is what indicates transitive ability, not a distance effect in the trials following transfer. For example, although beta*Q* rapidly manifests a symbolic distance effect over the first few dozen trials following transfer, accuracy *at* transfer remains quite poor (< 0.6). This is the value of the implicit inference stage: It permits a robust distance effect to emerge before any non-adjacent pairs have been trained. This suggests that subjects relied on serial representations, rather than the frequency with which stimuli are paired with rewards [27].

Betasort’s most salient discrepancy with the empirical data is its inability to correctly estimate adjacent pair accuracy during adjacent pair training: It overestimates the accuracy of monkeys during training, and underestimates that of humans. Although symbolic distance effects at transfer suggest that the model is on the right track, several of the model’s assumptions will need to be examined more deeply. It is likely, for example, that subjects adjust the explore/exploit tradeoff over time, which the model would represent by adjusting the values of *τ* and *ξ*. It is also plausible that the manner in which *τ* is has been implemented could be improved, to permit a more continuous range of possibilities between unbiased and random behavior. Although an improved fit can always be achieved by adding parameters [42], we prefer to reconsider the existing parameters in the interest of parsimony.

### Implications for Associative Models

Prediction error has long been a seen as a possible basis for ‘laws of behavior’ [43], an approach which has been elaborated by modern associative theory [44]. Temporal difference learning (TDL) builds on this tradition by using RPE to provide a satisfactory descriptive account of neural activity under various conditions [45, 46]. For these reasons, TDL is typically seen as a successor to the Rescorla-Wagner account of Pavlovian conditioning [47].

Despite the ubiquity of TI effects in vertebrate species [2, 16], RPE models are categorically unable to yield appropriate inferences. In Fig 4, *Q*/softmax gradually develops a robust distance effect, but only does so when trained with all pairs. This is because RPE models like *Q*/softmax use observed frequency of reward to predict the efficacy of future actions, and thus can only exceed chance when the training data yield differential reward probabilities.

Such models fail transitivity tests, and do not display symbolic distance effects, because non-terminal stimuli are rewarded equally during training. Although models have been proposed that overcome the limits of RPE in specific cases [48, 49], they remain vulnerable to bias. To illustrate this, Lazareva and Wasserman [12] trained pigeons and RPE models on adjacent pairs from the 5-item list ABCDE. They then presented massed trials of only the pair DE. Pigeons correctly identified *B* > *D*, but all RPE models either concluded that the value of stimulus *D* was larger than that of every other stimulus, or responded at chance levels. RPE models fail to learn correctly from massed trials because they are only adaptive in cases where expected value is a suitable proxy for behavior. Despite outperforming *Q*/softmax during the initial training, beta*Q* is also vulnerable to massed trials. Betasort’s implicit inference stage protects against this, as it allows generalization that is robust against varying base rates.

It is nevertheless not our aim to argue that betasort is *the* learning model used by subjects to solve transitive inference problems. Rather, published TI results strongly suggest that organisms make use of representations that have a spatial character, and that represented list members benefit from implicit updating. The failure of *Q*/softmax, on the other hand, reflects the more general conclusion that TI performance cannot be explained by associative strength alone.

Betasort not only performs transitive inference, but does so at low computational expense. This efficiency depends chiefly on the sample space used to encode stimulus positions. This places betasort within the cognitive tradition, in which TI is explained in terms of an organism’s ability to construct a representation of the ordering, which then serves as the basis for subsequent judgments [50].

### Implications for Machine Learning Approaches

There is presently a great deal of justified excitement over developments in statistical decision theory [51]. This formal approach to decision-making permits algorithms (or, more precisely, sets of algorithms) to discover not only the value of actions, but also the rules that govern when actions should be performed. By erecting a scaffolding of machine learning on the foundations of utility theory, algorithms may now be specified that can learn very complex tasks [23]. This approach also allows a wealth of new RL models to be formalized and considered.

Because of its reliance on machine learning formalisms, this modern branch of decision theory inherits much of the former’s terminology. “Reinforcement learning,” for example, is not only a class of behaviors displayed by organisms, whose operations we seek as scientists to discover. RL is sometimes put forward as a normative framework to explain how such learning takes place [54]. Although formal reinforcement learning is sometimes presented as a serviceable statistical model [18], other accounts assert [54] (or imply [55]) that the brain directly instantiates RL algorithms. Trying to relate betasort to this literature results in the seemingly contradictory statement that betasort is a (phenomenological) reinforcement learning model that does not rely on (formal) reinforcement learning.

The “model-based vs. model-free” distinction is also a source of terminological confusion. Model-free and model-based RL algorithms are often described as being analogous to associative and cognitive mechanisms, respectively [52, 53]. Neural signals that correlate with signals expected from ‘model-free” learning algorithms [56–58] or with ‘model-based’ algorithms [19, 60, 59] are sometimes presented as evidence that such algorithms are literally implemented, rather than merely being statistical models of associative and cognitive mechanisms respectively. Uncovering these signals is a major accomplishment for neuroscience. Nevertheless, many models that are distinct from machine learning methods might be responsible for these signals. Betasort is one such model; other include information-theoretic models [61], Bayesian models [62], or incentive salience models [63]. When reinforcement learning phenomena are framed exclusively in the language of machine learning, theory about the former becomes conflated with the limitations of the latter.

Betasort is not alone in its failure to map cleanly onto formal RL. Because TDL follows is an obvious successor to the Rescorla-Wagner model, Pavlovian models are generally considered “model-free.” However, many Pavlovian models require “model-based” techniques to be described in machine learning terms [64]. Because the “model-based” umbrella covers a wide variety of methods and implementations, it might be more fruitful to discuss the specific features of individual models than it is to attempt to classify them as “model-free” or not.

Considering a wider diversity of models facilitates this feature-based approach. Betasort, for example, violates the intuition that reinforcement learning of a cognitive map must necessarily be resource- or memory-intensive. What distinguishes it is its representation of uncertain position along a putative scale, and the use of implicit updating. An extensive empirical literature suggests that these are the features needed to perform TI, whether the algorithm can be described in terms of RL or not.

Machine learning’s rigorous formalism is both admirable and useful, but on its own, it represents a growing collection of tools. These tools will continue to fruitfully illuminate the hard problems of neural computation, but they should not be seen as the only tools. Phenomena such as TI suggest that abstract distinctions, such as those between associative learning and cognitive representation, remain fruitful for future theorizing about the brain (see, for example, Moses and colleagues [65]). It is time to wean ourselves of the habit of “confirming” specific models in the brain on the basis of neural activity correlated with some aspect of those models.

### Generality & Future Directions

Betasort is a specialized algorithm that relies upon strong assumptions. Meanwhile, *Q*/softmax is at a disadvantage because it is ill-suited to inferences of the sort TI requires. A good test of the spatial coding hypothesis, then, would be to implement different models with similar aims. The operations of betasort could, for example, be emulated using a POMDP model [66–69]. We hypothesize that such a model would display similar behavioral characteristics to betasort, *provided* such a model makes use of an instantiated linear state space that tracks putative stimulus position and uses implicit updating to modify all such position estimates.

Contrastingly, we predict that a POMDP that does not have these characteristics will fail to perform TI correctly, likely making the kinds of error observed in the case of beta*Q*. The reason for this prediction is subtle but important: Betasort succeeds at TI because it uses feedback to update a model of position; these are not the expected value of each stimulus. Instead, the value of a stimulus is a function of its relative position along a linear continuum. The adjacent-pair training method prevents the accurate convergence of RPE approximations, but does not prevent subjects (or betasort) from learning the ordering of the stimuli. A wealth of empirical evidence, particularly symbolic distance effects, suggest that a linear state space is essential. Additionally, massed trials will disrupt stimulus orderings unless some form of implicit updating is employed. At present, the literature is mute as to whether either an appropriately designed POMDP or the betasort algorithm can be instantiated in the brains of tilapia and tree shrews.

Another approach is to instantiate neural networks directly, emphasizing biological plausibility. Rombouts and colleagues [70, 71] report a neural network model that combines traditional RPE learning with global neuromodulation governed by integrating memory units. These integrators maintain representations of all stimuli, even those not presently visible. By comparing the model’s RPE learning to this sustained working memory representation, discrepancies can be detected and implicit inferences performed. Although Rombouts and colleagues have not yet demonstrated that their model can pass the critical test of transitivity (adjacent-only training, followed by testing on non-adjacent, non-terminal pairs), it nevertheless provides an example of how to construct a plausible computational model based on cognitive and associative mechanisms.

There is every indication that future iterations the betasort algorithm will handle a broad range of serial tasks. The simultaneous chaining procedure [13, 72] is another example of a task used extensively in the study of non-human serial learning [73]. During each trial, a set of images is presented, which must be selected in a specific order to yield a reward. Subjects must discover this response sequence by trial and error, using no cues other than the fact that the trial ends upon an incorrect choice.

Although simultaneous chain performance is difficult for associative models to account for [74], it is difficult for a different reason than TI. In TI, trials are logically ambiguous and the outcome provides incomplete information. That said, the configuration of stimuli in each trial has a discoverable correct answer. Solving a simultaneous chain, however, requires overcoming the *assignment of credit* problem [75]. Since all stimuli remain visible throughout the trial, the experiment does not provide external cues to guide behavior. It is therefore up to the subject to keep track of their progress in the list, and to treat progress as an implicitly informative cue.

Because betasort can select stimuli from sets of arbitrary size, simultaneous chains can be solved as a successive process of elimination. Completing a five-item simultaneous chain would require five iterations of the cycle depicted in Fig 2, with each success eliminating the selected item from the list of remaining candidates. An erroneous response would end the trial, and the algorithm would start over. The beta parameters learned from this process would then be available for use in other serial tasks, consistent with reports of transfer between serial tasks [13, 73].

The quest to decipher the brain’s cognitive machinery faces substantial obstacles. In the context of transitive inference and models like betasort, the clearest difficulty is implicit updating. As described, betasort updates every stimulus during every trial. This implies that, on any given trial, neural signals will be observed that relate to stimuli not presently visible. It is unclear how the presence of such implicit signals can be detected in either single-unit recordings or from fMRI data. Nevertheless, evidence from behavior suggests that such updating is likely taking place. The difficulty of detecting these implicit mechanisms is a challenge for recording techniques, not a weakness of cognitive theories. Until our understanding of brain networks advances to a stage that permits more comprehensive examination of the contents of memory, making theoretical commitments to specific mathematical formalisms hinders the discovery of other plausible accounts.

## Materials And Methods

### Monkey Experimental Procedures

#### Subjects

Subjects were three male rhesus macaques (Macaca mulatta). Subject treatment conformed with the guidelines set by the U.S. Department of Health and Human Services (National Institute of Health) for the care and use of laboratory animals. The study was approved by the Institutional Animal Care and Use Committee at Columbia University and the New York State Psychiatric Institute. Monkeys were prepared for experiments by surgical implantation of a post used for head restraint, and a scleral search coil [76]. All surgery was performed under general anesthesia (isoflurane 1–4%) and aseptic conditions. Monkeys were then trained using positive reinforcement to sit in a primate chair for the duration of the experiment with their heads restrained and to perform visual discrimination and eye movement tasks for liquid rewards while eye movements were recorded. Although subjects had extensive experience (> 6 months) with a version of the task during which all pairs were presented in a counterbalanced fashion (a procedure identical to that reported by Jensen and colleagues [13]), they were naive with respect to the adjacent-pair training procedure at the beginning of the experiment.

#### Apparatus

Subjects were seated in an upright primate chair while head movements were restrained by head post. Visual stimuli were generated by a VSG2/5 video controller (CRS, Cambridge, UK). The output from the video controller was displayed on a calibrated color monitor with a 60 Hz non-interlaced refresh rate. The spatial resolution of the display was 1280 pixels by 1024 lines. The video controller was programmed to send out digital pulses (frame sync) for timing purposes at the beginning of each video frame in which a stimulus was turned on or off. These pulses were recorded by the computer using a hardware timer and stored together with the eye movement data. Unless otherwise noted, the apparatus was identical to that described by Teichert and colleagues [77].

Eye position was recorded using a monocular scleral search coil system [76, 78] (CNC Engineering, Seattle, WA). Horizontal and vertical eye position signals were digitized with 12-bit resolution at a sampling rate of 1 KHz per channel. Eye velocity was computed offline by convolving eye position with a digital filter, constructed by taking the first derivative of a temporal Gaussian, *G*(*t*):

$$\frac{dG}{dt}=-k\cdot t\cdot \exp(\frac{-t^{2}}{\theta^{2}})$$ (13)

Here, *θ* = 8 msec, and *k* is a constant that sets the filter gain to 1.0. This filter does not introduce a time shift between the position input and velocity output, but adds temporal uncertainty to the velocity estimates. Horizontal eye velocities *h*′(*t*) and vertical eye velocities *v*′(*t*) were combined to estimate radial eye speed *r*′(*t*), where speed is the magnitude of the two-dimensional velocity vector:

$$r^{\prime }(t)=\sqrt{h^{\prime }{\left(t\right)}^{2}+v^{\prime }{\left(t\right)}^{2}}$$ (14)

Eye speed was used to estimate the onset of saccadic eye movements.

#### Procedure

A “session” is here defined as a continuous string of trials using a particular ordered list of seven photographic stimuli. In some cases, subjects performed more than one session during a day. A total of 107 sessions were completed across three animals (35 sessions, 51 sessions, and 21 sessions for the three subjects respectively).

Photographic stimuli were randomly selected in advance from a bank of 2500 stock photographs. The only criteria for assembling such a list was that stimuli were visually checked to ensure that they did not appear easy to confuse for one another. Although the list ordering was stored in the computer, it was never displayed or otherwise explicitly communicated to the monkey. By convention, we will refer to the seven items presented during a session using the letters A through G. Here, stimulus *A* is considered the “first” (i.e. “best”) list item and was always rewarded when selected, whereas stimulus *G* is considered the “last” (i.e. “worst”) list item and its selection was never rewarded. For the remaining stimuli (*B* through *F*), its selection was only rewarded when it held an earlier list position than any other visible stimulus.

Individual trials began with a central fixation point (0.5 deg red square) for 933 ms. Following fixation, two stimuli were presented on opposite sides of the central point. The fixation target disappeared at the same time the pictures appeared. Subjects responded by making a saccade to one stimulus or the other, and then fixating on that stimulus for 0.5 s, at which time feedback was provided. Failure to perform the initial fixation, or to saccade to a stimulus within 4 s of stimulus presentation, led to the trial being deemed “incomplete.” Once a saccade was made to the chosen stimulus, subjects were required to fixate for 1 s on the stimulus in order to receive a reward of 3 to 5 drops of juice. Number of drops varied day to day as a function of subject motivation, but was always held constant within a session.

Each session was divided into “blocks” of trials. A block consisted of randomly permuted presentations of a set of stimulus pairs, counterbalanced for screen position. For example, the six adjacent pairs in a session were the pairs AB, BC, CD, DE, EF, and FG. A block of adjacent pair trials would thus consist of twelve trials, with each pair presented twice (e.g. once with the on-screen arrangement AB, and once as BA). Subjects did not begin a new block until they had completed all trials in a previous block. If the monkey made an incorrect response, the trial was not repeated within the same block. In the event that a trial was deemed incomplete, another pair was randomly selected from the list of pairings not yet completed in that block. These steps ensured that there was equal information provided about all stimuli.

Each session began with an adjacent-pair training phase, subjects completed 20 blocks consisting of only the six adjacent pairs (240 trials total). Subjects then completed an additional ten blocks (counterbalanced for position and presented without replacement) of all twenty-one pairs (420 trials total). Throughout the session, the only information subjects received about the stimuli were the rewards (or lack thereof) during completed trials; at no point were they shown all stimuli at once, nor were there position cues suggesting stimulus ordering. In general, responding resembled that seen when macaques perform TI using a touchscreen apparatus (Jensen et al., 2013), suggesting that performance was modality-independent.

### Human Experimental Procedures

#### Participants

Nineteen college undergraduate volunteers gave written consent to participate in the experiment for course credit. The study was approved the Institutional Review Board in the Human Research Protection Office of Columbia University.

#### Apparatus

Participants selected stimuli by touching them using a touchscreen (Keytec, Inc.) mounted on a 15 in deskmiddle computer monitor. Unless otherwise noted, the apparatus was identical to that described by Merritt and Terrace [79].

#### Procedure

To introduce them to the task, participants first completed a session with a list of seven novel images, consisting of 4 blocks (168 trials) of all pairs. Positive feedback was indicated with a magenta screen and a bell sound, and negative feedback was indicated with a black screen and a whoosh sound. Feedback was always provided immediately following each touch. Apart from being told how to distinguish positive vs. negative feedback, and an instruction to “do as well as you can,” participants were given no further instruction regarding the objectives or content of the task.

After this practice session, participants immediately completed a second session with a new list of seven items, also lasting 168 trials. They first completed 3 blocks (36 trials) of only the adjacent pairs, then completed 3 blocks (90 trials) of only the non-adjacent pairs. Finally, they completed a single block (42 trials) of all pairs.

## Supporting Information

S1 Text Pseudocode for computational models. Detailed pseudocode describing the computational workings of the betasort, beta*Q*, and *Q*/softmax algorithms.
(PDF)

S1 Dataset TI behavior data. Raw data of choices made by rhesus macaques and human participants during sessions of the TI task.
(ZIP)
