# Supplementary material for: Implicit Value Updating Explains Transitive Inference Performance: The Betasort Model
Source: PLoS Comput Biol. 2015 Sep 25;11(9):e1004523. doi: 10.1371/journal.pcbi.1004523 (PMC4583549; doi:10.1371/journal.pcbi.1004523)
Supplement: S1 Text — Detailed pseudocode describing the computational workings of the betasort, betaQ, and Q/softmax algorithms. (PDF) [file pcbi.1004523.s001.pdf]

Implicit value updating explains transitive inference performance: The betasort model (Supplemental Information)

Greg Jensen[1,2*], Fabian Muñoz[1], Yelda Alkan[1], Vincent P. Ferrera[1,3], Herb Terrace[2]

Algorithm 1: The betasort updating policy.

Data: memory arrays **U**, **L**, **R**, **N**, chosen stimulus ch, unchosen stimulus nc, outcome r, recall ξ
Result: updated model **U**, **L**, **R**, **N**

```
begin
    R ← R · ξ; N ← N · ξ                          /* Relax R and N */
    E ← R/(R + N)                 /* Estimate trial reward rates */
    ξ_R ← E/(E + 1) + 0.5
    U ← U · ξ_R · ξ; L ← L · ξ_R · ξ              /* Relax U and L */
    V ← U/(U + L)                 /* Estimate stimulus positions */
    if r = 1 then
        U ← U + V; L ← L + (1 − V)  /* consolidate all stimuli */
    else
        U_nc ← U_nc + 1                              /* shift nc up */
        L_ch ← L_ch + 1                            /* shift ch down */
        for j = 1 to 7 do
            if j ≠ ch AND j ≠ nc then
                if V_j > V_ch AND V_j < V_nc then
                    U_j ← U_j + V_j; L_j ← L_j + (1 − V_j)   /* consolidate j
                    */
                else if V_j < V_nc then
                    L_j ← L_j + 1                   /* shift j down */
                else if V_j > V_ch then
                    U_j ← U_j + 1                     /* shift j up */
    return U, L, R, N
```

Algorithm 2: The beta$Q$ updating policy.

Data: memory arrays $\mathbf{U}$, $\mathbf{L}$, $\mathbf{R}$, $\mathbf{N}$, chosen stimulus ch, unchosen stimulus nc, outcome r, recall ξ

Result: updated model $\mathbf{U}$, $\mathbf{L}$, $\mathbf{R}$, $\mathbf{N}$

begin

- $R_{ch} \leftarrow R_{ch} \cdot \xi$; $R_{nc} \leftarrow R_{nc} \cdot \xi$ `/* Relax` R_{ch} `and` R_{nc} `*/`
- $N_{ch} \leftarrow N_{ch} \cdot \xi$; $N_{nc} \leftarrow N_{nc} \cdot \xi$ `/* Relax` N_{ch} `and` N_{nc} `*/`
- $\mathbf{E} \leftarrow \mathbf{R}/(\mathbf{R} + \mathbf{N})$ `/* Estimate trial reward rates */`
- $\boldsymbol{\xi}_{\mathbf{R}} \leftarrow \mathbf{E}/(\mathbf{E} + 1) + 0.5$
- $U_{ch} \leftarrow U_{ch} \cdot \boldsymbol{\xi}_{\mathbf{R}_{ch}} \cdot \xi$; $U_{nc} \leftarrow U_{nc} \cdot \boldsymbol{\xi}_{\mathbf{R}_{nc}} \cdot \xi$ `/* Relax` U_{ch} `and` U_{nc} `*/`
- $L_{ch} \leftarrow L_{ch} \cdot \boldsymbol{\xi}_{\mathbf{R}_{ch}} \cdot \xi$; $L_{nc} \leftarrow L_{nc} \cdot \boldsymbol{\xi}_{\mathbf{R}_{nc}} \cdot \xi$ `/* Relax` L_{ch} `and` L_{nc} `*/`
- $\mathbf{V} \leftarrow \mathbf{U}/(\mathbf{U} + \mathbf{L})$ `/* Estimate stimulus positions */`
- **if** $r = 1$ **then**
  - $U_{ch} \leftarrow U_{ch} + V_{ch}$; $L_{ch} \leftarrow L_{ch} + (1 - V_{ch})$ `/* consolidate` ch `*/`
  - $U_{nc} \leftarrow U_{nc} + V_{nc}$; $L_{nc} \leftarrow L_{nc} + (1 - V_{nc})$ `/* consolidate` nc `*/`
- **else**
  - $U_{nc} \leftarrow U_{nc} + 1$ `/* shift` nc `up */`
  - $L_{ch} \leftarrow L_{ch} + 1$ `/* shift` ch `down */`
- **return** $\mathbf{U}$, $\mathbf{L}$, $\mathbf{R}$, $\mathbf{N}$

Algorithm 3: The Q-learning updating policy.

Data: memory array $\mathbf{Q}$, chosen stimulus ch, unchosen stimulus nc, outcome r, modifier α

Result: updated model $\mathbf{Q}$

begin

- **if** $r = 1$ **then**
  - $Q_{ch} \leftarrow Q_{ch} + \alpha \cdot (1 - Q_{ch})$ `/* shift` ch `up */`
  - $Q_{nc} \leftarrow Q_{nc} - \alpha \cdot Q_{nc}$ `/* shift` nc `down */`
- **else if** $r = 0$ **then**
  - $Q_{ch} \leftarrow Q_{ch} - \alpha \cdot Q_{ch}$ `/* shift` ch `down */`
  - $Q_{nc} \leftarrow Q_{nc} + \alpha \cdot (1 - Q_{nc})$ `/* shift` nc `up */`
- **return** $\mathbf{Q}$
